# Cation-Controlled Assembly, Activity, and Organization of Biomimetic DNA Receptors in Synthetic Cell Membranes

**DOI:** 10.1021/jacs.5c09928

**Published:** 2025-09-06

**Authors:** Elita Peters, Diana A. Tanase, Lorenzo Di Michele, Roger Rubio-Sánchez

**Affiliations:** † Department of Chemical Engineering and Biotechnology, 2152 University of Cambridge, Philippa Fawcett Drive, Cambridge CB3 0AS, U.K.; ‡ Department of Chemistry, Molecular Sciences Research Hub, Imperial College London, London W12 0BZ, U.K.; ¶ fabriCELL, Molecular Sciences Research Hub, Imperial College London, London W12 0BZ, U.K.

## Abstract

Biological cells use cations as signaling messengers to regulate a variety of responses. Linking cations to the functionality of synthetic membranes is thus crucial to engineering advanced biomimetic agents such as synthetic cells. Here, we introduce bioinspired DNA-based receptors that exploit noncanonical G-quadruplexes for cation-actuated structural and functional responses in synthetic lipid membranes. Membrane confinement grants cation-dependent control over receptor assembly and, when supplemented with hemin cofactors, their peroxidase DNAzyme activity. Cation-mediated control extends to receptor lateral distribution to localize DNA-based catalysis within phase-separated membrane domains of model synthetic cells, imitating the localization of multimeric membrane complexes to signaling hubs in living cells. Our modular strategy paves the way for engineering from the bottom-up cation-responsive pathways for sensing, signaling, and communication in synthetic cellular systems.

## Graphical abstract

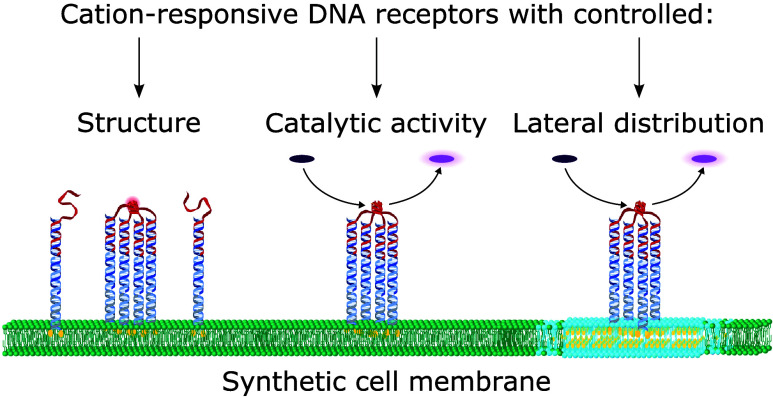

## Introduction

Cell membranes possess specialized mechanisms to detect and transduce environmental cues to coordinate cellular responses.^1^ Signal transduction pathways commonly rely on membrane receptors assembling multimeric complexes and/or undergoing conformational changes, which trigger downstream functionality through membrane-hosted reactions, e.g., (de)­phosphorylation.^2^ Cations, like calcium^3,4^ and potassium^5^ ions, are central to cellular signaling, typically shuttled across biological membranes as messengers to link physicochemical stimuli to responses as varied as synaptic activation,^6^ muscle contraction,^7^ and cellular motility.^8^

Synthetic cell science aims to build cell-like agents that replicate some of the intricate behaviors observed in living matter.^9,10^ Synthetic cells thus provide bottom-up platforms to dissect fundamental biological processes^11^ and to unlock disruptive applications in healthcare and biotechnology.^12^ By borrowing concepts and building blocks from biological and inorganic sources, solutions to engineer synthetic cells have achieved a wide range of life-like functionalities, from reconstituting cytoskeletal support^13–15^ to sustaining directional motion^16,17^ to membrane-based energy harvesting^18^ and mechanochemical sensing.^19^

Particularly promising for the construction of human-made mimics of cells are the tools of nucleic acid nanotechnology,^20,21^ as they provide robust design rules to engineer both structural and functional elements of synthetic cells. DNA and RNA nanostructures have been developed as replicas of cytoskeletal fibers^22,23^ and membrane-less organelles,^24–27^ or interfaced with lipid membranes.^28,29^ Amphiphilic DNA nanostructures have been applied to synthetic cell membranes to program several biomimetic responses,^30–32^ ranging from molecular sensing^33^ to tissue formation^34,35^ to surface^36,37^ and transmembrane^38–40^ transport to bilayer remodelling^41,42^ and vesicular fission pathways.^43^

Given the central role of cation homeostasis in health and disease, biomimetic devices that grant structural and functional responsiveness to cations are highly desirable, as they could help realize the fundamental and applied potential of synthetic cells. However, despite recent developments,^44–46^ pathways for embedding responsiveness to cations in bioinspired systems, and particularly synthetic cell membranes, are still scarce. G-quadruplexes (G4s) are cation-stabilized noncanonical secondary structures, studied in biology for their role in transcription–translation to regulate gene expression, telomere maintenance, and disease progression.^47–51^ In the context of DNA nanotechnology, G4s are convenient structural and responsive motifs^52–54^ for membrane bioengineering, as they have also been coupled to lipophilic groups to develop pathways for cation transmembrane transport.^55–57^

Here, we introduce biomimetic DNA-based membrane receptors with structural and functional responsiveness to physiologically relevant cations. By interfacing cholesterol-modified DNA nanostructures with intermolecular G-quadruplexes, we present a membrane bioengineering strategy to modulate the formation of receptors on the surface of lipid bilayers through independent design criteria, namely, the guanine content and choice of cationic conditions. We supplemented our cation-controlled receptors with hemin cofactors to host and regulate DNAzyme peroxidase reactions on lipid membranes. Finally, we exploit DNA receptor assembly to localize their peroxidase activity within lipid domains of phase-separated synthetic cell membranes. Our modular platform thus enables cation-actuated structural and functional responses in the membranes of synthetic cellular agents, paving the way for sophisticated biomimetic platforms toward bottom-up membrane signaling, molecular transport, and division.

## Results and Discussion

### G4s Assemble Cation-Stabilized DNA Membrane Receptors

Our DNA nanodevices consist of 56 base-pair (bp) long duplexes composed of four synthetic DNA oligonucleotides (Tables S1 and S2), featuring a terminal overhang with six consecutive guanines, (G)_6_, that allow for receptor assembly via an intermolecular G-quadruplex.^44^

As depicted in Figure 1a, we produced ∼100 nm large unilamellar vesicles (LUVs) with dipalmitoylphosphatidylcholine (DOPC) lipids and decorated their membranes with our nanostructures, which host double-cholesterol (dC) “anchors” able to insert into the hydrophobic core of the bilayer.^60^ Membrane functionalization was carried out in buffered solutions containing 1× TE + 100 mM KCl + 87 mM sucrose, thus favoring G-quadruplex formation (see Experimental Methods section in the Supporting Information). Dynamic light scattering (DLS) measurements in Figure 1b at the various LUV functionalization stages show the expected gradual increase in hydrodynamic diameter resulting from the membrane-tethered DNA nanostructures with and without G-rich strands. When featuring G-rich strands, however, vesicle size distributions exhibit modest broadening, likely due to a low degree of LUV–LUV association mediated by intermembrane G-quadruplex formation. As seen in Figure S1, polydispersity indices below 0.2 indicate that LUV dispersions remain predominantly monodisperse with no large-scale vesicle aggregation. Note that, owing to the flexible spacer (nine thymines) separating G-runs from the double-stranded membrane anchors, G-quadruplex folding is not expected to impose significant conformational strain. This applies whether the four devices are tethered to the same membrane or to different vesicles. We therefore do not anticipate a meaningful energetic penalty or strain associated with G-quadruplex formation on the surface of a single LUV. Instead, kinetic effects are likely to play a more prominent role. At the experimental concentrations of LUVs and DNA nanostructures, we expect the rate of encounter to be much higher for DNA within the same membrane than across different LUVs, owing to the much higher local concentration of membrane-bound DNA relative to LUVs in solution, despite their similar diffusion coefficients (LUVs: *D* ∼ 2–4 μm^2^ s^–1^ and membrane-anchored DNA: *D* ∼ 1–5 μm^2^ s^–1^ ^61,62^ ). This suggests that binding rates will favor intra-LUV binding and thus G-quadruplexes to more likely assemble within the same vesicle membrane than across separate vesicles. We thus conclude that intramembrane G-quadruplex formation is the predominant mode of receptor assembly.

**Figure 1:**
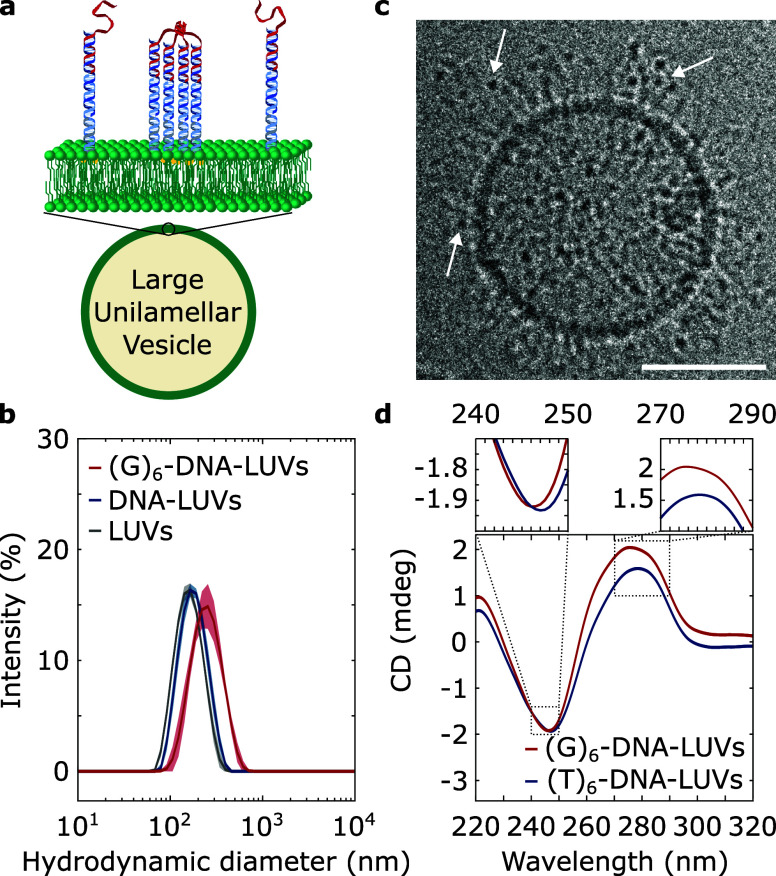
Guanine repeats in membrane-anchored DNA nanodevices guide the assembly of cation-stabilized receptors through G-quadruplexes. (a) Schematic depiction of lipid bilayers functionalized with DNA nanostructures featuring a G-rich overhang that assembles into intermolecular G-quadruplexes stabilized by cations. (b) Mean hydrodynamic diameter ± standard deviation of *n* = 3 measurements obtained through dynamic light scattering of large unilamellar vesicles (LUVs) lacking or featuring DNA nanodevices with or without the G-rich strand that supports G-quadruplex formation. (c) Cryo-electron micrograph of representative LUV functionalized with DNA nanodevices linked to G-quadruplexes, which appear as electron-dense regions^58^ (highlighted with white arrows). Scale bar: 50 nm. (d) Circular dichroism (CD) spectra of LUVs decorated with DNA nanostructures featuring either (G)_6_-overhangs (red) or a (T)_6_-repeat in place of the G-rich sequence (blue).

Cryo-electron microscopy, as seen in representative micrographs in Figures 1c and S2, shows membrane-bound G-quadruplexes, which manifest as electron-dense regions^58^ that are absent in LUVs decorated with nanodevices lacking the G-rich strand (Figure S3). Circular dichroism (CD) spectra of (G)_6_-DNA-decorated LUVs show peak shifts toward characteristic maximum (λ ≈ 263 nm) and minimum (λ ≈ 245 nm) wavelengths (Figure 1d), relative to LUVs decorated with DNA nanostructures in which the G-rich sequence is replaced with a poly thymine, (T)_6_-DNA. While the observed shift is moderate—likely due to the coexistence and different relative concentrations of G4s with double-stranded DNA anchors^63^—the overall spectral shift and curve shape are consistent with parallel G-quadruplex topologies^64^ and with the formation of tetramolecular receptors on the membrane surface.^65^ To confirm receptor stoichiometry, we introduced a toehold domain on the G-rich strand to enable detachment of the nanodevices from membranes with a strand displacement reaction.^66^ Following membrane functionalization and receptor assembly, we recovered the nanostructures from LUVs and conducted a gel-shift assay, comparing their electrophoretic mobility with that of tri-, tetra-, and pentavalent DNA junctions with arm lengths matching the size of a detached monomeric nanostructure. As shown in Figure S4, receptors exhibit migration profiles similar to those of tetravalent (4-way) junctions, confirming their tetramolecular nature. The slight reduction in mobility relative to 4-way junctions is likely due to the flexible linker, which is absent from the central junction of the control multivalent nanostructures.

### Cation-Controlled Receptor Assembly

Having assembled receptors by cross-linking individual DNA-based membrane inclusions via G-quadruplexes, we modified our nanostructure design and functionalization conditions to control G4 formation. Specifically, we tune the length of guanine repeats and identity of the cations, both known to alter the stability of G4s.^59^

We functionalized LUVs with DNA nanodevices featuring overhang variants (G)_*n*=4,5,6_ (Figure 2a) in buffered solutions with added physiologically relevant monovalent (K^+^, Na^+^, Li^+^) or divalent (Ca^2+^, Mg^2+^) cations (see Experimental Methods section and Table S3 summarizing cation concentration ranges in biological environments^67^). To assess G-quadruplex formation, we exploited the fluorescence enhancement of *N*-methyl mesoporphyrin IX (NMM), which selectively binds to parallel G-tetrads.^44,68^ Fluorescence emission spectra in Figures S5 and S6 demonstrate that different cationic environments do not induce noticeable peak shifting. In turn, Figure S7 shows that, relative to baseline values in our experimental buffers, NMM exhibits a moderate fluorescence increase in the presence of control (T)_6_-DNA nanostructures, likely due to weak nonspecific interactions, which also depend on the cationic environment. As schematically depicted in Figure 2b, we used NMM fluorimetry to compare the presence of membrane-bound G-quadruplexes with G4s assembled in the bulk from nanostructures at a nominally equal concentration ([(G)*_n_*] = 0.4 μM, at a DNA/lipid molar ratio of ∼8 × 10^–4^). To that end and to ensure nanostructures remained fully dispersed in solution, we used noncholesterolised DNA constructs. Cholesterol-bearing DNA nanostructures undergo micellization in aqueous environments,^69,70^ and those would provide locally increased concentrations that would obscure the specific contributions of membrane confinement to receptor assembly. Indeed, owing to their tetrameric stoichiometry,^65^ oligonucleotide concentration is expected to have a large impact on the formation of intermolecular G4s. We therefore anticipate differences in the abundance of G-quadruplexes in the bulk relative to those tethered to LUVs, where confinement to membrane surfaces increases the effective local nanostructure concentration.

**Figure 2:**
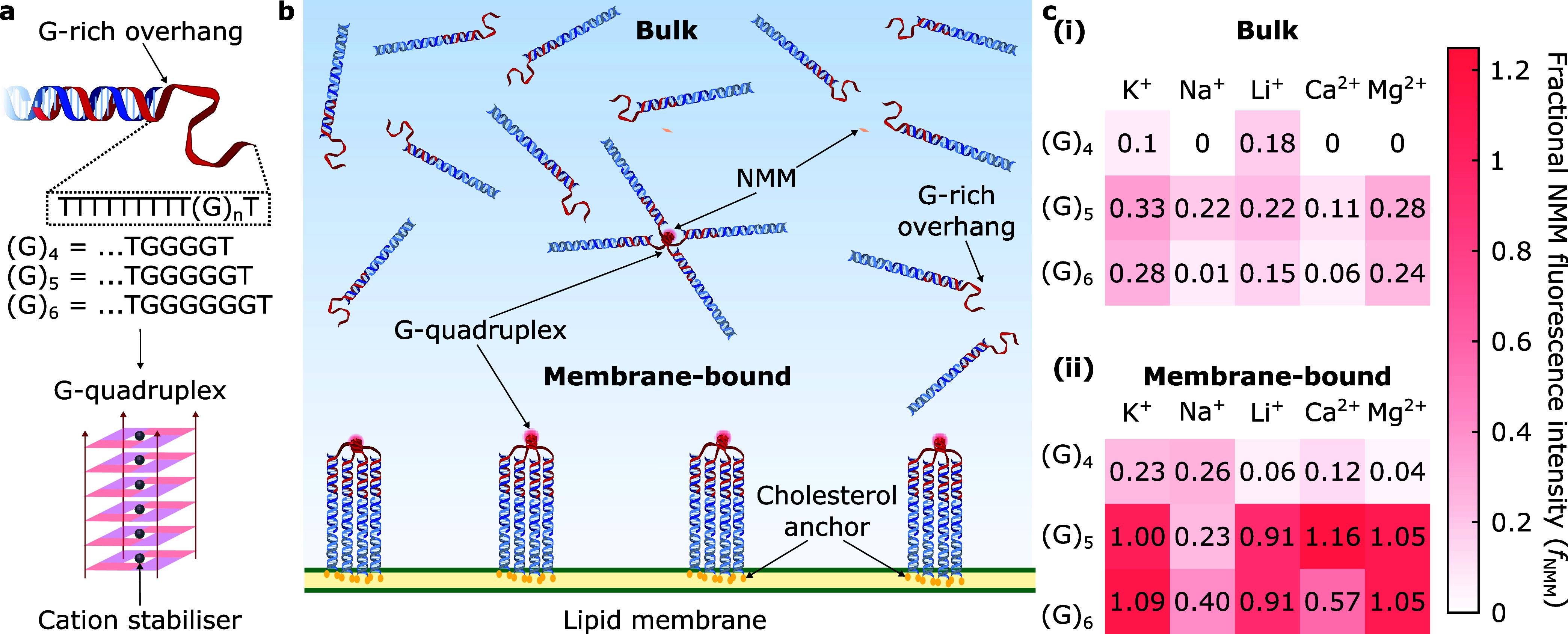
Membrane confinement grants control over G-quadruplex formation. (a) Schematic representation of the G-rich overhang (in red) linked to DNA nanodevices, highlighting the sequence for each design variant (G_4_, G_5_, and G_6_), which can assemble into intermolecular, parallel G-quadruplexes stabilized by cations. The schematic is illustrative, and cation coordination sites can vary (e.g., interplanar vs in-plane) depending on cation size.^59^ (b) Graphical depiction of tetramolecular receptors assembled through G-quadruplexes in the bulk and when membrane-bound via double-cholesterol anchors. Parallel G-tetrads selectively bind NMM and enhance its fluorescence, enabling us to monitor the abundance of G-quadruplexes. (c) Fractional NMM fluorescence intensity (*f*_NMM_) heatmaps (see Figure S11 for the associated standard deviation δ*f*_NMM_) summarizing the relative abundances of receptors in (i) the bulk and (ii) membrane-bound as a function of monovalent (K^+^, Na^+^, or Li^+^) and divalent (Ca^2+^ or Mg^2+^) cations for designs G_4_, G_5_, and G_6_ (at a nominal concentration of [(G*_n_*)] = 0.4 μM).

To quantify the relative abundance of G-quadruplexes, we compute a fractional NMM fluorescence intensity (*f*_NMM_), defined as the cation-specific background-subtracted average intensity of DNA-decorated LUVs normalized by the NMM fluorescence intensity of (G)_5_-DNA-LUVs in K^+^ (see Experimental Methods section). The latter was chosen as it is the condition in which we found the highest fraction of G4s when nanostructures were allowed to assemble in the bulk via thermal annealing at high concentrations ([(G)*_n_*] = 6 μM), as quantified with agarose gel electrophoresis (Figure S8) and observed with NMM fluorimetry (Figure S9). Note that representative, time-invariant fluorescent profiles in Figure S10 confirm that both G-quadruplex formation and NMM stacking onto G-tetrads reached thermodynamic equilibrium.

When the nanostructures were dispersed in the bulk, *f*_NMM_ values ≤0.33 shown in Figure 2c,i (and their comparatively high standard deviation, δ*f*_NMM_, in Figure S11) suggest low G4 abundance relative to DNA-decorated LUVs. Indeed, tethering our nanodevices to lipid membranes, and thus increasing their effective local concentration, led to systematically higher *f*_NMM_ values across the tested G-run lengths and cationic conditions (Figure 2c,ii), confirming the expected effect on G4 assembly. The shortest G-repeat, (G)_4_, with low *f*_NMM_ values—albeit higher than their bulk counterparts (with the exception of Li^+^)—has relatively low G-quadruplex abundances compared to LUVs decorated with (G)_5_ in KCl, where *f*_NMM_ ≡ 1. CD signatures of (G)_4_-decorated LUVs in K^+^ show slight characteristic peak shifts (Figure S12), consistent with the parallel quadruplex topologies observed for LUVs carrying (G)_6_- (Figure 1c) and (G)_5_-overhangs (Figure S13). Note that while the combination of CD spectroscopy, NMM fluorimetry, and gel-shift assays indicates the formation of parallel, tetramolecular, G-quadruplexes, the number of stacked tetrads in G4s cannot be unambiguously assigned without high-resolution crystallographic data. We speculate, however, that the tier number should be equal to the length of the G-run.^71^

Various cationic compositions, namely, Li^+^ and Mg^2+^, enable G4 formation in (G)_5_ and (G)_6_ constructs to a comparable degree to that of (G)_5_-membranes with K^+^ (reaching *f*_NMM_ ∼ 1 and within the experimental error in Figure S11). Similarly, (G)_5_-DNA-decorated LUVs in Ca^2+^ have *f*_NMM_ values moderately higher than 1, suggesting favorable G4 formation. Interestingly, (G)_6_- and (G)_5_-runs in the presence of Na^+^ show the lowest relative abundance, with *f*_NMM_ ∼ 0.40 and ∼0.23, respectively.

To substantiate our hypothesis that the higher local DNA concentrations achieved with membrane confinement influence G-quadruplex formation and stability, we performed simple numerical calculations using a thermodynamic description of tetramolecular G4 assembly from four monomeric DNA constructs (see Supporting Information Note I). Briefly, we relate the fraction of DNA in G4 constructs (*p*) with the total DNA concentration (*C*) and the standard free energy of assembly (Δ*G*°), capturing the sharp changes of *p* with increasing DNA concentrations (Figure S14). As an example, in the case of Δ*G*° = −30*k*_B_ *T*, low DNA concentrations, such as those used in our bulk assembly assays, yield negligible probabilities of G4 assembly *p*. In contrast, when membrane-confined, the estimated local concentration (∼171 μM; see Supporting Information Note II) yields *p* ∼ 0.7, corresponding to a 14-fold increase in G-quadruplex formation probability.

While the specific free energies of G4 formation in our experiments are not known, the numerical solutions indicate that the high local DNA concentrations on membranes can substantially increase the probability of G-quadruplex assembly, even with ions that, like lithium, do not stabilize G4s in the bulk. Thus, our calculations provide a thermodynamic rationale for how membrane attachment, by increasing the local concentration of DNA, shifts the equilibrium in favor of G4 assembly even in the presence of weakly coordinating cations. Notably, molecular dynamic simulations show that Li^+^ can indeed weakly coordinate with G-tetrads, albeit with free energies of bond formation ∼15 kcal mol^–1^ less favorable than K^+^ ions.^72^ We thus argue that, despite weak coordination of Li^+^, under membrane confinement, where DNA is locally concentrated, G-quadruplex formation probabilities would be substantially higher.

Furthermore, in view of the higher G4-stabilization expected to occur with Na^+^ cations compared to Mg^2+^ or Li^+^, the trends on G4 stability seen for DNA-LUVs (Figure 2c,ii) could also be influenced by the interplay between DNA–DNA and DNA–lipid interactions. Indeed, each cation species has a distinct affinity for phosphate groups present on both DNA and phosphatidylcholine (PC) lipid headgroups. Cations have been shown on DNA–lipid systems to screen Coulomb interactions and/or mediate bridging to different extents, thus inducing differences in the membrane attachment of DNA nanostructures.^45^ It is thus possible, for instance, that divalent cations further increase nanostructure effective concentration (and favor G4 formation) by bridging membranes with DNA duplexes.^45,46^

To gain insight into the assembly dynamics of our receptors, we decorated LUV membranes with nanodevices in the presence of KCl but lacking the G-rich strand, thus preventing G4s. We subsequently added a mixture of stoichiometrically adjusted (G)_6_- or (G)_5_-strands and NMM, allowing oligonucleotides to rapidly diffuse, hybridize with membrane-bound nanostructures, and assemble into K^+^-stabilized G-quadruplexes where NMM could stack, leading to fluorescence enhancement (see Experimental Methods section). Consistent with the high local concentrations of DNA nanostructures upon membrane confinement (∼171 μM), NMM fluorescent profiles in Figures S15 and S16 suggest fast G-quadruplex assembly. Equilibration of fluorescence under 250 s for both design variants is likely limited by the diffusion of single-stranded oligonucleotides throughout the samples, as we expect the diffusion time scales of free oligonucleotides in solution (at the relevant concentrations and lengths)^73^ to be on the order of hundreds of seconds.

### Cation-Actuated DNA Receptors with Membrane-Hosted Catalytic Activity

Our platform to control the formation of cation-stabilized receptors can be linked to the functionality of DNAzymes underpinned by G4s. A prominent example is that of the horseradish peroxidase (HRP)-mimicking DNAzyme, composed of hemin cofactors stacked onto G-tetrads.^74^ Binding to G4s strongly enhances the catalytic activity of hemin when converting AmplexRed (AR) to fluorescent resorufin in the presence of hydrogen peroxide (H_2_O_2_).^75^

We thus produced LUVs decorated with our nanodevices and incubated them with hemin cofactors, resulting in receptor assembly hosting the HRP-mimicking DNAzyme (Figure 3a). As shown in Figure 3b and S17, S18, we monitored resorufin production by means of fluorescence spectroscopy. In view of the trends summarized in Figure 2, we expect our various designs and cationic conditions to result in different amounts of receptors available for hemin to bind, and thus the various conditions to produce differences in peroxidation rates.

**Figure 3:**
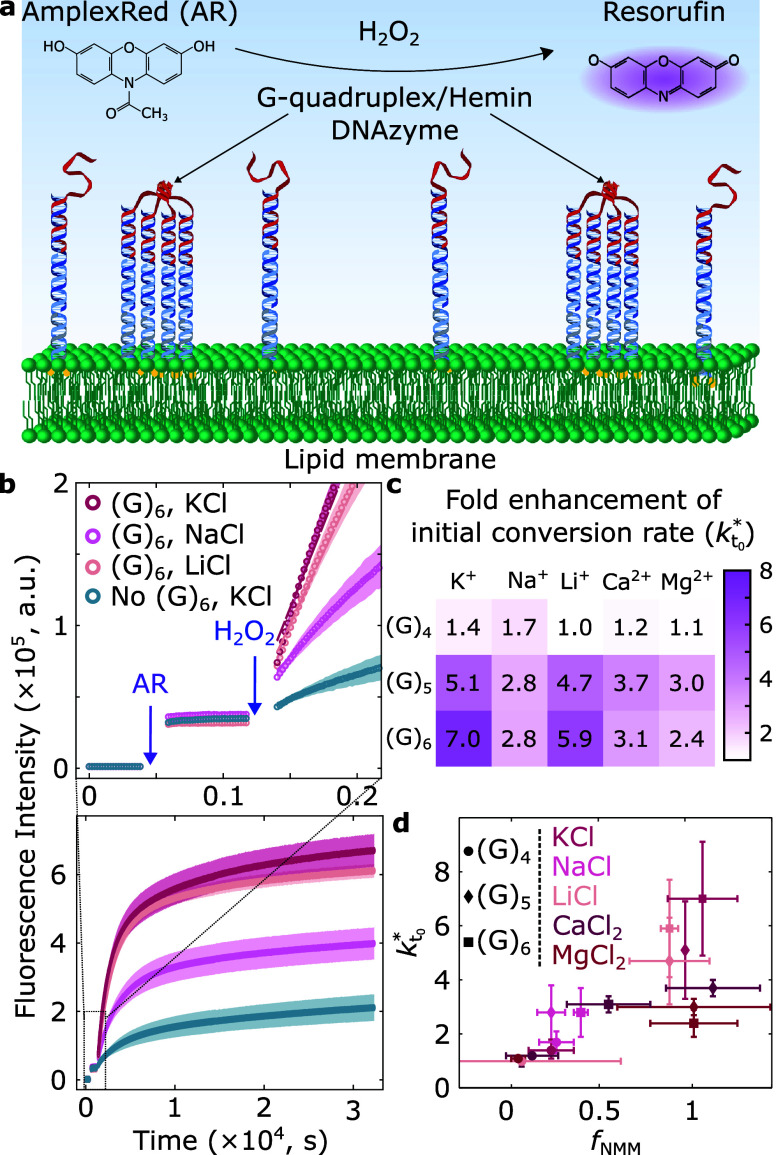
Cation-controlled DNA receptors for membrane-hosted peroxidase activity. (a) Schematic showing the incorporation of hemin cofactors to form the horseradish peroxidase mimicking DNAzyme, which in the presence of hydrogen peroxide (H_2_O_2_) catalyzes the conversion of AmplexRed (AR) to fluorescent resorufin. (b) Representative resorufin fluorescence intensity profiles of DNA-decorated LUVs with and without (G)_6_-rich strands and added hemin cofactor in KCl, NaCl, and LiCl. The inset shows, at early times, the fluorescence intensity of LUVs before/after AR addition and its increase upon initiating the reaction with H_2_O_2_. Solid lines are linear fits used to extract the initial reaction rates (*k*_*t*_0__). (c) Heatmap of fold change in initial reaction rate (*k*_*t*_0__^*^ = *k*_*t*_0_,DNAzyme_/*k*_*t*_0_,hemin_) for receptors assembled from either (G)_*n*=4,5,6_ as a function of cationic conditions. (d) Rate fold changes (*k*_*t*_0__^*^) correlate with receptor relative abundance (*f*_NMM_); statistical significance assessed via Spearman test: ρ = 0.7876, *p*-value = 4.89 × 10^–4^.

To quantify the influence of our assembly platform on resorufin production, we extracted the initial reaction rates (*k*_*t*_0__) from linear fitting of the fluorescent traces (see inset Figure 3b and Experimental Methods section) and computed their fold enhancement relative to DNA-LUVs (i.e., lacking G4s) in the presence of hemin cofactors using *k*_*t*_0__^*^ = *k*_*t*_0_,DNAzyme_/*k*_*t*_0_,hemin_. To rule out the possibility that nonspecific interactions between hemin with duplexes or unstructured DNA could contribute to catalysis, we compared peroxidation across (G)_6_-DNA-LUVs, (T)_6_-DNA-LUVs, DNA-LUVs, and nonfunctionalized LUVs. As shown in Figure S19, control conditions exhibit largely overlapping behaviors, confirming that, in our platform, hemin catalytic activity is enhanced in the presence of G-quadruplexes.

The heatmap in Figure 3c (and the associated propagated uncertainty δ*k*_*t*_0__^*^ in Figure S20) readily confirms that various conversion rates can be accessed by design. Note that, consistent with our observations on relative G4 abundance in Figure 2c,ii, LUVs functionalized in the presence of LiCl result in higher *k*_*t*_0__^*^ values compared to NaCl. We thus further explored the relationship between relative receptor abundance and fold change in the initial peroxidation rate (*k*_*t*_0__^*^ vs *f*_NMM_). As shown in Figure 3d, we find a statistically significant correlation (nonparametric statistical correlation Spearman test: ρ = 0.7876; *p*-value = 4.89 × 10^–4^), with greater fold changes corresponding to higher *f*_NMM_ values. Small deviations to this trend emerge for specific conditions with divalent cations, which, despite reaching high *f*_NMM_ values, exhibit moderately lower fold-changes in the peroxidation rate. We ascribe this decrease to a lower catalytic efficiency of hemin in the presence of divalent cations, as seen in the fluorescent traces in Figures S17 and S18, where peroxidation is catalyzed solely by the cofactor. Importantly, although a minor inter-LUV association was observed with DLS in Figures 1b and S1, we do not expect clustering to substantially alter DNAzyme functional performance, given that hemin cofactors and the reaction substrates (AmplexRed and H_2_O_2_) are small molecules that could still freely diffuse through LUV–LUV contacts.

### Localizing Receptor Activity in Membrane Domains of Synthetic Cells

The cation-dependent assembly and activity of our DNA receptors can be readily coupled to membrane phase separation. Their synergy enables to mimic the reorganization and activity of multimeric protein nanomachines within cellular membrane domains.

To exemplify the applicability of our platform for synthetic cell engineering, we produced giant unilamellar vesicles (GUVs) as the chassis of our model synthetic cells and decorated their membrane with our receptors. Synthetic cells, prepared from lipid mixtures containing 1,2-dioleoyl-phosphatidylcholine (DOPC)/1,2-dipalmitoyl-phosphatidylcholine (DPPC)/cholesterol in 2:2:1 molar ratios, displayed membrane phase separation at room temperature with coexisting liquid-ordered (*L*_o_) and liquid-disordered (*L*_d_) domains.^77^ A fluorescent TexasRed-DHPE lipid marker was included to stain *L*_d_ phases, while our DNA nanodevices were modified to include a fluorescein probe to monitor their lateral organization by means of confocal microscopy.

Given the partitioning of double-cholesterol anchors to lipid domains in phase-separated membranes,^36,78,79^ DNA receptor assembly enriched *L*_o_ domains by cross-linking four sets of dC anchors, as shown schematically in Figure 4a and with representative confocal micrographs in Figure 4b. Segmentation of confocal equatorial micrographs allowed us to assess DNA nanostructure partitioning by comparing average fluorescence intensities in *L*_o_ and *L*_d_ phases (see Supporting Note III). Expectedly, as seen in Figure S21, monomeric DNA nanodevices (i.e., lacking the G-rich strand) moderately accumulate in *L*_o_ domains. Subtle differences in their *L*_o_-partitioning tendencies already emerged between added salts, further supporting our hypothesis of cations modulating electrostatic DNA–DNA and DNA–lipid interactions. Stronger partitioning of dC-anchored nanostructures is achieved in the presence of divalent cations, while Li^+^ promotes greater *L*_o_-accumulation when compared to Na^+^ and K^+^. These differences can be ascribed to variations in screening effectively the negatively charged phosphate groups present both on the DNA backbone and the lipid headgroups on the membrane surface.^45^

**Figure 4:**
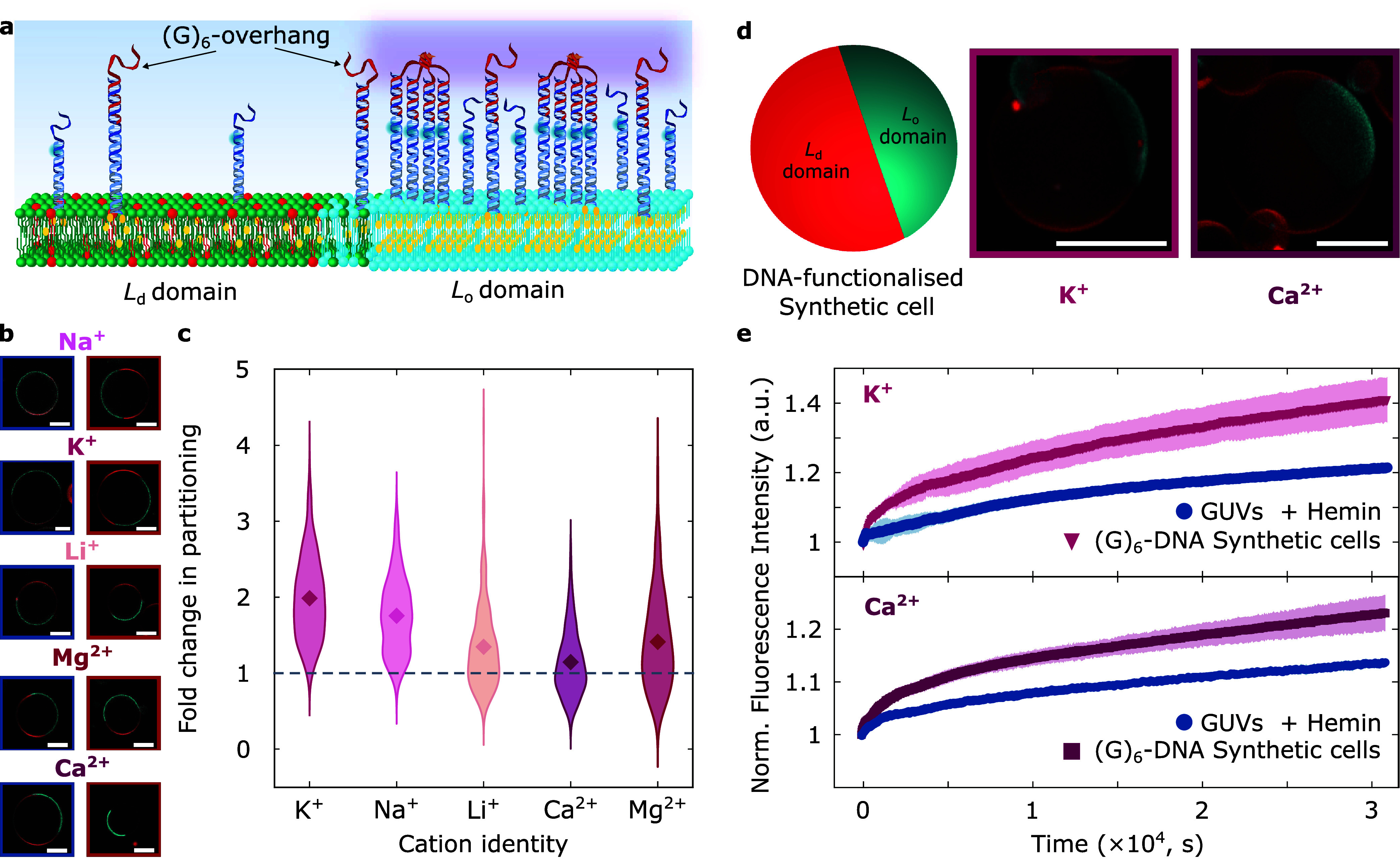
Cation-dependent assembly localizes receptors and their peroxidase activity in lipid domains of synthetic cell membranes. (a) Schematic of the organization in lipid domains of DNA nanodevices with and without (G)_6_-strands, and therefore, the possibility of receptor assembly localizing peroxidase activity when supplemented with hemin cofactors. (b) Representative confocal equatorial micrographs of GUVs functionalized with DNA nanodevices lacking (left, framed in blue) and featuring (right, framed in red) (G)_6_-strands to support receptor formation in buffered solutions with added monovalent (Na^+^, K^+^, or Li^+^) or divalent (Mg^2+^, or Ca^2+^) salts. The TexasRed-DHPE signal, staining the *L*_d_ phase, is shown in red, while that of fluorescein (FAM)-labeled DNA nanostructures is shown in cyan. (c) Violin plots of the fold change in *L*_o_-partitioning (*K*_p_^*^), computed from confocal micrographs (see Experimental Methods section) of DNA-decorated GUVs in either monovalent (Na^+^, K^+^, or Li^+^) or divalent (Mg^2+^, or Ca^2+^) cationic conditions, showing the cation-dependent membrane distribution of DNA receptors. Diamonds are mean fold change values, while the dashed line denotes no change (*K*_p_^*^ = 1). (d) Schematic depiction of a DNA-decorated synthetic cell (left) and three-dimensional (3D) views of reconstructions (obtained from Volume Viewer, FIJI^76^) from confocal z-stacks of representative DNA-decorated GUVs (in the presence of K^+^ or Ca^2+^) as synthetic cell models with *L*_o_-localized functionality. (e) Normalized fluorescence profiles monitoring resorufin production from DNA-functionalized synthetic cells in the presence of K^+^ (top) or Ca^2+^ (bottom) relative to GUVs lacking DNA functionalization but supplemented with the hemin cofactor, showing the possibility to localize peroxidase activity in lipid domains of synthetic cell membranes. All scale bars are 10 μm.

Conversely, when nanostructures feature the (G)_6_-overhang, thus enabling G4 formation, devices systematically show stronger partitioning relative to their states prior to the addition of the G-rich strand. In Figure 4c we summarize the fold enhancement in *L*_o_-partitioning (*K*_p_^*^) of (G)_6_-DNA membranes against that of functionalized GUVs lacking (G)_6_-overhangs. Fold changes, with statistically significant values of *K*_p_^*^ > 1 (*p* < 1.2 × 10^–16^; one-tailed nonparametric Wilcoxon signed-ranked test—see Table S4 for individual *p*-values) are indicative of stronger affinities for *L*_o_ domains due to the increased number of cholesterol anchors per nanostructure.^36^ The latter therefore supports the presence of tetramolecular receptors and highlights our ability to regulate their membrane distribution in a cation-dependent fashion. Notably, due to the macroscopic sizes of GUVs, membrane curvature effects in domain partitioning are expected to be negligible, as the membrane can be considered to be locally flat relative to the size of the nanodevices. This interpretation is further supported by the absence of noticeable correlation between domain accumulation and vesicle size across different cationic compositions (Figure S22 and Table S5 with nonparametric Spearman ρ and *p-*values).

We subsequently sought to localize receptor peroxidase activity in *L*_o_ domains of our synthetic cells. As a proof-of-concept, we selected K^+^ or Ca^2+^ as monovalent or divalent cation messengers, respectively, and incubated DNA-decorated synthetic cells (shown in Figures 4d and S23 with representative 3D views from confocal z-stacks) with hemin cofactors. We monitored resorufin production upon triggering synthetic cell activity with H_2_O_2_, as summarized in Figure 4e with normalized fluorescent profiles (as well as on Figures S24 and S25 with individual replicate profiles), where expectedly, synthetic cell membranes featuring our receptors exhibit faster and higher conversion than membranes lacking functionalization in the presence of the hemin cofactor. Epi-fluorescence micrographs in Figure S26 of synthetic cells after peroxidation confirm their stability throughout the experimental time scales. While direct imaging of resorufin being produced at lipid domains is limited by diffusion time scales, our results showing the preferential accumulation of membrane-bound, catalytically active receptors in liquid-ordered phases strongly suggest that catalytic activity is predominantly localized in *L*_o_-domains. Therefore, with our platform, we show the possibility to localize and regulate activity within membrane domains of synthetic cells using different cation messengers.

## Conclusions and Outlook

In summary, we have introduced biomimetic receptors with cation-controlled assembly, catalytic activity, and distribution in synthetic cell membranes. Our modular platform affords stimulus-responsive assembly of catalytically active receptors that sustain lateral reshuffling on membrane surfaces, integrating cation-responsiveness with spatial organization and catalytic function. Our tetrameric membrane receptors exploit the formation of intermolecular G-quadruplexes to assemble on the surface of lipid bilayers. By exploiting the increased effective local concentrations induced by membrane confinement, we show that receptor formation can be controlled by different cations. Similarly, cation-mediated control allows us to regulate the rate of membrane-hosted reactions, as exemplified with peroxidation using the HRP-mimicking DNAzyme. Finally, by interfacing our cation-actuated receptors with phase-separated membranes in GUVs, we established a link between the presence of cations and the lateral organization of functional DNA-based membrane inclusions. We applied our nanodevices to host peroxidase activity within lipid domains of model synthetic cells, thereby imitating the ability of cells to localize reactions within cell-membrane domains as hypothesized to occur during signaling cascades.^80,81^

Our strategy has direct applications in bottom-up synthetic biology to engineer advanced synthetic cell models. Because of its modularity and the versatility of DNA nanotechnology, our cation-responsive approach to cross-link individual membrane inclusions can be readily adapted to guide the assembly of more intricate DNA and RNA origami nanodevices. Indeed, simple design changes can enable coupling the presence of cations to the formation of cortex-like platforms^82,83^ and membrane-remodelling nanostructures,^42,43^ unlocking the development of cation-dependent pathways for cellular motion, trafficking, division, and bioinspired synaptic transmission. Similarly, the ionic environments assessed in our work are within cation concentration ranges relevant to in vitro transcription and cell-free expression systems,^84,85^ suggesting possible synergies with the functionality of our receptors for synthetic cell engineering. In addition, the possibility of destabilizing G-quadruplexes (e.g., with photoinduced damage^86,87^ or chelating agents^88^) paves the way for the development of sophisticated dynamic behaviors that respond to both physical and chemical stimuli.

Similarly, our DNA receptors could underpin the investigation of fundamental biological processes. One can envision our nanodevices as probes^89^ tethered to cellular membranes that could report on changes in electrochemical activity, allowing, for instance, monitoring the kinetics of cation waves^90,91^ as well as cation transport across biomembranes.^92–94^

Finally, the effect of surface confinement on local concentration, as exploited here to regulate peroxidase activity, could be leveraged to influence the action of other DNAzymes, ribozymes^95^ and (split) aptamers,^96^ many of which are typically underpinned by G4s. For instance, it could be possible to finely tune the efficiency of Mg-dependent cleaving nanostructures^97^ or the allosteric modulation of enzymatic action,^98^ thereby unlocking the possibility to host in (bio)­membranes a wider range of reactions useful in biosensing, biotechnology, and bioengineering.

## Supplementary Material



## Notes

Experimental methods, supporting notes, supporting figures, supporting table with cation concentrations in physiological environments, supporting tables with statistical significance and correlation values, and DNA sequences of the nanostructures used throughout this work (PDF)
